# In Vitro Antibacterial Effect of the Methanolic Extract of the Korean Soybean Fermented Product Doenjang against *Staphylococcus aureus*

**DOI:** 10.3390/ani11082319

**Published:** 2021-08-05

**Authors:** Klara Lalouckova, Lucie Mala, Petr Marsik, Eva Skrivanova

**Affiliations:** 1 Department of Microbiology, Nutrition and Dietetics, Faculty of Agrobiology, Food and Natural Resources, Czech University of Life Sciences Prague, Kamycka 129, 165 00 Prague, Czech Republic; lalouckova@af.czu.cz (K.L.); malalucie@af.czu.cz (L.M.); 2 Department of Nutritional Physiology and Animal Product Quality, Institute of Animal Science, Pratelstvi 815, 104 00 Prague, Czech Republic; 3 Department of Food Science, Faculty of Agrobiology, Food and Natural Resources, Czech University of Life Sciences Prague, Kamycka 129, 165 00 Prague, Czech Republic; marsik@af.czu.cz

**Keywords:** inhibition, antibiotics, pathogens, microbiota, resistance

## Abstract

**Simple Summary:**

The emergence of bacterial antibiotic resistance is a negative phenomenon occurring worldwide in both animals and humans. The EU banned the use of antibiotic growth promoters in animal production, as their administration to livestock is assumed to substantially contribute to the spread of bacterial resistance. Therefore, alternatives to antibiotic substances are needed to maintain the quality and quantity of animal products. Certain plant materials, such as fermented soybean products, can serve as a source of substances with potential to decrease the growth of resistant bacteria, such as *Staphylococcus aureus*. Fermented soybean products, including doenjang, are known to contain natural phytoestrogens called isoflavones, which are especially interesting due to their antimicrobial activity; these products can also be utilized in animal feed. Thus, the antibacterial activity of the methanolic extract of the Korean soybean fermented product doenjang was evaluated using standardized microbiological methods against nine strains of resistant and sensitive *S. aureus*, including those occurring in animals. The extract has been shown to be active at a concentration range of 2048–4096 µg/mL against all tested *S. aureus* strains and can therefore serve as a promising alternative to antibiotics in animal feed after additional testing in the laboratory and on living animals.

**Abstract:**

Ultra-high performance liquid chromatography/mass spectrometry showed soyasaponin I and the isoflavones daidzein, genistein, and glycitein to be the main components of the methanolic extract of the Korean soybean fermented product doenjang, which is known to be a rich source of naturally occurring bioactive substances, at average contents of 515.40, 236.30, 131.23, and 29.00 ng/mg, respectively. The antimicrobial activity of the methanolic extract of doenjang against nine *Staphylococcus* *aureus* strains was determined in vitro by the broth microdilution method to investigate its potential to serve as an alternative antibacterial compound. The results suggest that the extract is an effective antistaphylococcal agent at concentrations of 2048–4096 µg/mL. Moreover, the tested extract also showed the ability to inhibit the growth of both methicillin-sensitive and methicillin-resistant animal and clinical *S. aureus* isolates. The growth kinetics of the chosen strains of *S. aureus* at the minimum inhibitory concentration of the methanolic extract of doenjang support the idea that the tested extract acts as an antibacterial compound. To the best of our knowledge, this is the first report on the antistaphylococcal action of the methanolic extract of doenjang thus, additional studies including in vivo testing are necessary to confirm this hypothesis.

## 1. Introduction

Staphylococcal infections are among the most widely spread global public health problems of recent times [1]. These infections are caused by *Staphylococcus aureus*, a commensal and opportunistic pathogen that is the leading cause of bacteremia, endocarditis, skin and soft tissue infections, bone and joint infections, and hospital-acquired infections in humans [2], and they are also of serious concern in livestock animals as they negatively influence animal production [3], are transmittable from animals to humans [4], can cause food poisoning [5], and their occurrence is connected with the potential risk of development of bacterial antibiotic resistance [6]. Thus, *S. aureus* represents a serious public health burden in both hospital and community settings as well as an economic and animal welfare problem, especially in dairy farming [7]. Moreover, importance of combating *S. aureus* is not only limited to serious diseases and economic losses but includes also its spread in the environment. Infections in animals are deleterious to animal health, and animals can act as a reservoir for staphylococcal transmission to humans [8]. Nowadays, the percentage of *S. aureus* isolates reported as methicillin-resistant *S. aureus* (MRSA), e.g., in Southern and Eastern Europe, is higher than 25% [9]. The median value of the number of people infected with methicillin-resistant *S. aureus* (MRSA) in the European Union/European Economic Area was around 150,000 in 2015, with an estimated 7000 of people dying from the disease [10]. Antibiotic-resistant *S. aureus* was observed within 1 year of the first clinical use of semisynthetic antistaphylococcal penicillins, which were developed in approximately 1960 [11], and since then, both MRSA and methicillin-sensitive *S. aureus* (MSSA) strains have gained resistance to a wider variety of regularly used antimicrobials [12,13]. According to Turner et al. [14], much of the genetic diversity of MRSA and other pathogens occurs within the accessory genome consisting of mobile genetic elements such as pathogenicity islands, bacteriophages, chromosomal cassettes, transposons, and plasmids, which are acquired by horizontal transfer between strains, and where mediators of virulence, immune evasion, and antibiotic resistance are commonly found. Another feature increasing the ability of *S. aureus* to resist antibacterial treatment is biofilm formation on various materials. Biofilms act as protective sites, where the bacterium is relatively shielded from the action of antimicrobial agents and the host immune response [15].

With the aim of reducing the spread of antimicrobial resistance, EC Regulation No. 1831/2003, in effect since 2006, banned the use of antibiotic growth promoters in livestock production among the European Union countries. Following the introduction of this regulation, concerns were raised regarding decreased animal production and the quality and safety of livestock products; consequently, widespread interest in alternative antimicrobials has grown [16].

Currently, natural substances still play a highly significant role in the discovery and development process for new drugs [17] because natural products are a rich source of compounds with antibacterial activity [18]. A significant percentage of new antibiotics are either natural products themselves or are derived from natural products [19]. In many cases, natural alternatives in the form of unique bioactive compounds can be found in microorganisms, plants, and animal species that thrive in extreme environments such as rainforests, deserts, and hot springs [20]. To maintain the safe and sufficient production of animal-originating products, various kinds of alternative non-antibiotic growth promoters have been suggested by different authors. These include phytogenic additives such as cinnamon [21], thyme [22], or black cumin [23]; essential oils such as oregano [24] or peppermint [25]; probiotics [26]; synbiotics [27]; clay minerals [28]; and organic acids [29].

Among other plant species, soybean (*Glycine max*) is a rich source of bioactive compounds, including isoflavones [30], which are active secondary metabolites synthesized during the initial growth stages of soybeans and are present mainly in the cotyledon and hypocotyl of soybean seeds [31]. Isoflavones comprise two different chemical forms, known as aglycones and glucosides, which are both found in soybean and soy foods [32]. The content of isoflavones and other active components in soybean is influenced by various factors, including climate, genotype, growing season, water availability, and location [33]. Soybeans are a fundamental element of the human diet in Asian countries and are commonly used to produce fermented foods such as doenjang (soybean paste) [34]. Traditionally, doenjang is produced during the fermentation of previously modified soybeans by naturally occurring bacteria (such as *Bacillus subtilis*) and fungi (e.g., *Aspergillus* spp., *Penicillium* spp., *Mucor* spp., and *Rhizopus* spp.) [35]. Studies have proven the effect of microbial fermentation on the increase in the proportion of aglycones by cleavage of the β-glycosyl bond of isoflavone glucoside, providing an advantage over nonfermented foods by improving biological activity [36]. The main isoflavones of soybean in aglycon form are daidzein, genistein, and glycitein [37]. The antimicrobial properties of the doenjang isoflavones against a wide variety of bacteria and bacterial biofilms have been previously described by various authors. However, this information is limited mainly to the activity of the pure isoflavones. Genistein, the most studied soy-isoflavone, is known to inhibit the growth of *Bacillus anthracis*, *B. cereus*, *Helicobacter pylori,* MRSA, *Streptococcus pyogenes*, and *Vibrio harveyi* at concentration of 27 µg/mL but is not effective against *Escherichia coli*, *Klebsiella pneumoniae, Lactobacillus reuteri*, and *Shigella sonnei* [38,39]. However, results vary between authors. For example, Hummelova et al. [40] observed antibacterial action of genistein at a concentration of ≤128 µg/mL against *B. cereus* and *Streptococcus pyogenes* but not against *Enterococcus faecalis, Listeria monocytogenes, S. aureus,* and *S. epidermidis*. According to Ulanowska et al. [41], daidzein, another soy-isoflavone, inhibited the growth of *Pseudomonas aeruginosa* (concentration ≥ 2.5 µg/mL), *V. harveyi* (concentration ≥ 7.5 µg/mL), *Citrobacter freundii*, *Micrococcus luteus* (concentration 7.5 µg/mL), and *Sarcina* sp. (concentration 25 µg/mL) but did not inhibit the growth of *Bacillus subtilis*, *S. aureus*, *E. coli*, *K. pneumoniae*, *Proteus vulgaris*, *Salmonella enterica*, and *Serratia marcescens*.

In addition to isoflavones, soybeans are known to contain other bioactive molecules such as saponins, peptides, lecithins, protease inhibitors, or phytosterols [30,42], some of which can contribute to the antibacterial action of soybean, its products, or their extracts. Nevertheless, other biologically active substances or nutrients may influence the antimicrobial activity of the studied compounds originating in soybeans. For instance, according to Hassan et al. [43], the soybean saponin-rich methanolic extract had no antibacterial activity against *S. aureus*, *Salmonella typhimurium*, and *E. coli* at concentrations ≤12,500 µg/mL.

In addition to antibacterial effects, regular intake of doenjang has been reported to suppress body weight gain [44,45] and to improve blood pressure [46], cytokine levels [47], and serum oxidative stress [48]. Compared to other soybean fermented products, doenjang is a more promising source of isoflavones because of its higher proportion of free to glycoside-bound isoflavones [49].

To the best of our knowledge, there are no studies on the antibacterial activity of the methanolic extract of doenjang (MED), meaning the mixture of doenjang bioactive compounds that are soluble in methanol, against *S. aureus* strains—especially those strains causing diseases in livestock. Therefore, this study was performed to evaluate the antibacterial effect of the MED against both MSSA and MRSA strains that are commensals or pathogens of humans and/or animals.

## 2. Materials and Methods

### 2.1. Methanol Extraction of Isoflavones and Soyasaponins from the Fermented Soybean Paste Doenjang

A fermented soybean paste (doenjang) sample from Haechandle (Daejeon, South Korea) weighing 100 g was extracted for 24 h at room temperature in 100 mL of methanol (Sigma Aldrich, Prague, Czech Republic) on a laboratory shaker (GFL, Burgwedel, Germany) at 200 rpm. The extract was then filtered and concentrated using a rotary evaporator (Büch Labortechnik, Flawil, Switzerland) under vacuum at 40 °C and subsequently lyophilized. The dried doenjang residues were then dissolved in dimethyl sulfoxide (DMSO; Sigma Aldrich, Prague, Czech Republic) as a stock solution with a concentration of 51,200 µg/mL and stored at −20 °C until use. The final DMSO concentration in the samples did not exceed 1% and thus did not influence the results of antibacterial effect testing [50]. The sample was weighed before and after lyophilization to determine the overall yield of the extraction.

### 2.2. Ultra-High Performance Liquid Chromatography/Mass Spectrometry of Isoflavones and Soyasaponins in the MED

Analytical standards of isoflavonoids (all with purity of at least 98%) were purchased from INDOFINE Chemical Company Inc. (Hillsborough, NJ, US), and a standard of soyasaponin I (purity ≥94%) was obtained from Sigma-Aldrich (Prague, Czech Republic). Isoflavones and soyasaponins were analyzed on a UHPLC/MS-HRAM system consisting of an Ultimate 3000 (Thermo Fisher Scientific, Waltham, MA, US) chromatograph and a high-resolution accurate mass (HRAM) Q-TOF mass spectrometer Impact II (Bruker Daltonics, Berlin, Germany). Compounds were separated on an Acclaim RSLC 120 C18 column (2.2 µm, 2.1 × 100 mm; Thermo Fisher Scientific, Waltham, MA, US) using gradient elution with mobile phases consisting of 0.2% formic acid (A) and methanol (B). The gradient started at 2% B (0 to 2 min) followed by an increase to 100% B in 15 min, was then kept at 100% B for 20 min, and was finally returned to 2% B in 21 min and equilibrated at this concentration before the next injection to 27 min. The column temperature was 35 °C and the flow rate of the mobile phase was 250 µL/min. The injection volume was 5 µL. Analytes were detected in positive ionization mode using an ESI ion source (for details of the settings, see Table S1). Tentative identification of glycoside conjugates of isoflavones as well as several soyasaponins was carried out based on their exact mass and isotopic pattern in combination with analysis of fragmentation spectra obtained at four collision energy levels (15, 20, 30, and 40 eV) in MS/MS experiments (see Figures S1 and S2 and Table S2). Samples were prepared in three dilutions: 2, 1, and 0.5 µg/mL in 50% MeOH. Calibration samples for isoflavones and soyasaponin I quantitation were prepared at concentrations of 5, 10, 25, 50, 100, 250, and 500 ng/mL. Quality control (QC) samples were prepared as a mixture of standards at a concentration of 100 ng/mL in a mobile phase and were applied after each five injections with the exception of calibration samples. Extract samples were measured in five repetitions. Limits of detection (LODs) and limits of quantitation (LOQs) were estimated as the concentration corresponding to three-fold and ten-fold, respectively, the noise in the extract sample. Acquisition of mass spectrometry data was carried out using oTof Control 4.0 and HyStar 3.2 software (Bruker Daltonics, Berlin, Germany). Qualitative and quantitative analyses were performed using DataAnalysis 4.3 and TASQ 4 software (both Bruker Daltonics, Berlin, Germany), respectively. Annotation of fragmentation spectra was performed using Mass Frontier 7.0.5.9 SR3 (High Chem Ltd., Bratislava, Slovakia).

### 2.3. Bacterial Strains and Their Maintenance

In total, nine bacterial *S. aureus* strains, both type cultures and clinical isolates, were tested in this study. The sources of the bacterial strains included the American Type Culture Collection (ATCC; Manassas, VA, USA) and the Czech Collection of Microorganisms (CCM; Brno, Czech Republic). The strains (Table 1) used were MSSA ATCC 29213, CCM 4442, and CCM 6188 and MRSA ATCC 33591, ATCC 43300, and ATCC BAA 976. Two clinical drug-resistant isolates of *S. aureus* (SA 2, and SA 3) and one epidemic MRSA strain (EMRSA-15) were obtained from the Motol University Hospital (Prague, Czech Republic). Based on the previously determined MICs values [51,52,53], the clinical isolates were identified to be resistant as follows: SA 2—resistant to oxacillin (MIC 68 µg/mL), gentamicin (MIC 16 µg/mL), and tetracycline (MIC 8 µg/mL); SA 3—resistant to gentamicin (MIC 8 µg/mL), and penicillin (MIC 18.67 µg/mL); EMRSA-15—resistant to oxacillin (MIC 99.56 µg/mL), and penicillin (MIC 16 µg/mL). The clinical isolates were identified using matrix-assisted laser desorption/ionization time-of-flight mass spectrometry (MALDI-TOF MS) as described previously [51]. Bacterial aliquots were stored at −80 °C in a mixture of Mueller—Hinton broth (Oxoid, Basingstoke, UK) and 20% glycerol, except for CCM strains, which were stored in a mixture of tryptone soya broth (Oxoid, Basingstoke, UK) and 20% glycerol. Working cultures were maintained in Mueller—Hinton or tryptone soya broth at 37 °C for 24 h prior to testing.

### 2.4. Testing the Antibacterial Effects of the MED

In vitro broth microdilution was performed in a 96-well microtiter plate using the guidelines of the Clinical and Laboratory Standards Institute [54] and the latest findings [55] to determine the minimum inhibitory concentrations (MICs) of the MED. A descending two-fold serial dilution of stock solution in the appropriate nutrient medium (90 µL) was performed in the microtiter plate, starting at a concentration of 16,384 µg/mL. A suitable bacteriological broth was used according to the bacterial strain tested. The bacterial suspension was standardized to a density of 5 × 10^5^ CFU/mL by calibrating the turbidity of the bacterial inoculum to 0.5 on the McFarland scale and adding an amount of 10 µL into wells. The bacterial plates were then incubated for 24 h at 37 °C.

### 2.5. Determination of the Minimum Inhibitory Concentration of Methanolic Extract Obtained from Doenjang

The growth of microorganisms in the medium was evaluated by measuring the turbidity in the individual wells using an Infinite^®^ 200 PRO Microplate Reader (Tecan, Männedorf, Switzerland) at a wavelength of 405 nm, as proposed by Cos et al. [55] for natural extracts. The MICs of the MED were evaluated as the lowest recorded concentrations of extract that caused a ≥80% decrease in bacterial growth in the wells compared to growth in the extract-free broth. Proper control of bacterial growth was evaluated by determining the susceptibility of the tested bacteria to penicillin G. To exclude the effect of DMSO on bacterial growth, its MICs were determined with a starting concentration of 10,000 µg/mL (1%). The MICs of the antibiotic and DMSO in the microtiter plates were determined as they were when testing the MICs of the MED. To confirm the data from MIC testing, 25 µL of methylthiazolyl diphenyltetrazolium bromide (MTT; Sigma Aldrich, Prague, Czech Republic) was pipetted into all wells of a microtiter plate after the incubation. All samples were tested in triplicate and in three independent experiments thus, the final MIC for every bacterium is a mode of nine values.

### 2.6. Evaluation of Bacterial Growth under Various Concentrations of the MED

To evaluate the influence of the MED concentration (MIC and ½ MIC) on the growth of different *S. aureus* strains, representative of the MSSA (ATCC 29213) and MRSA groups (ATCC 43300), a clinical specimen from the culture collection (ATCC BAA 976), and a clinical isolate obtained from Motol University Hospital in Prague, Czech Republic (SA 2), were chosen to undergo standardized microdilution assay along with growth curve modeling [56]. Briefly, determination was performed in 96-well microtiter plates, involving eight two-fold serial dilutions of the tested compound starting at a concentration of 4096 µg/mL (from which only the MIC and ½ MIC were chosen to be displayed in the growth curve figures) in Mueller–Hinton broth. Next, the plates were inoculated with bacterial suspensions at a final density of 5 × 10^5^ CFU/mL and, standardized using Densi-La-Meter II by adjusting the turbidity of the microorganism suspension to the 0.5 McFarland standard, as with the MIC determination. Subsequently, incubation at 37 °C was performed, and the absorbance of each well was measured spectrophotometrically by an Infinite^®^ 200 PRO Microplate Reader (Tecan, Männedorf, Switzerland) at 405 nm every fifteen minutes for 5.25 h, then every thirty minutes for 9 h, and additionally after 24 h.

## 3. Results

### 3.1. Qualitative and Quantitative Profile of Isoflavones and Soyasaponins Detected in the MED

**Figure 1:**
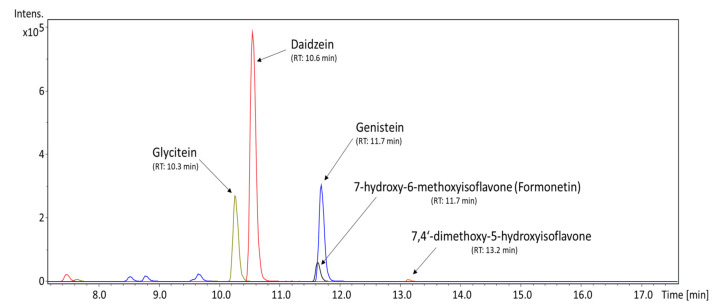
Extracted ion chromatogram of isoflavones from the methanol extract of doenjang, represented as m/z of [M + H]^+^.

UHPLC/MS analysis was used to determine the content of isoflavones and soyasaponins in the MED with regard to their possible antibacterial properties. As described in Table 2, the only isoflavones detected in countable values were the aglycone forms, namely daidzein, genistein, and glycitein, with respective amounts of 236.30, 131.23, and 29.00 ng/mg. Figure 1 shows their occurrence in the MED within a chromatogram. Other isoflavones that can contribute to the MED’s properties but that were present only as traces were demethyltexasin, dimethylgenistein, methoxyisoflavone, formonetin, 7-hydroxy-6-methoxyisoflavone, and 7,3′4′-trihydroxyisoflavone. 

Three isoflavone glycosides were found in the MED sample as well. They were tentatively identified as hexosyl derivatives of genistein (probably genistin), daidzein (daidzin), and glycitein (glycitin) (see Figures S1 and S2). However, according to the signal intensity of these compounds, their concentration in the sample was relatively low.

**Figure 2:**
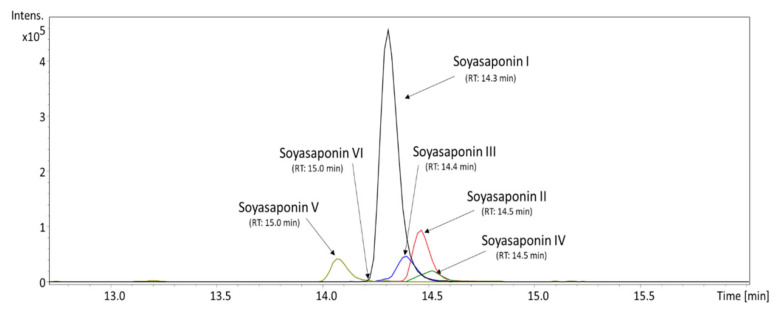
Extracted ion chromatogram of soyasaponins from the methanol extract of doenjang, represented as *m*/*z* of [M + H]^+^.

As described in Table 2, the most abundant soyasaponin found in the MED was soyasaponin I, with an average content of 515.40 ng/mL. In addition to soyasaponin I, five other minor soyasaponins were determined in the sample. They were tentatively identified as soyasaponin II, III, IV, V, and VI (see Figure S1 and Table S2). Other detected soyasaponins’ content was only estimated relatively as the percentage of the peak area of soyasaponin I due to the lack of reliable standards in the study. In terms of abundance in the sample, soyasaponin I was followed by soyasaponin II (0.189%), soyasaponin III (0.136%), soyasaponin V (0.111%), and soyasaponin IV (0.062%). The presence of soyasaponin VI was detected only in trace amounts, as can be seen in Figure 2.

The overall yield of the doenjang methanolic extraction was evaluated as the weight of the sample before and after lyophilization, with the amount of extracted material equal to 11.574 g in 100 g of the original sample, meaning that the fermented soybean paste doenjang contained 11.574% of substances used for antibacterial testing.

### 3.2. Antistaphylococcal Action of MED

Based on the results of the in vitro broth microdilution method, the growth of all nine tested *S. aureus* strains (both MSSA and MRSA) was inhibited by the methanolic extract of the Korean fermented soybean paste doenjang. As shown in Table 3, the MICs ranged from 2048 to 4096 µg/mL. The methanolic extract of the soybean fermented product doenjang shows an antibacterial effect against methicillin-sensitive and methicillin-resistant strains of *S. aureus*.

The most sensitive to the antibacterial activity of the MED were three *S. aureus* strains, namely ATCC 29213 (MSSA), the bovine mastitis isolate CCM 4442 (MSSA), and the clinical isolate SA 2 (MRSA), the growth of which was inhibited at a concentration of 2048 µg/mL. Two out of the three strains that were inhibited at a concentration of 2048 µg/mL were methicillin-sensitive. At a concentration of the MED of 2048 µg/mL, bioactive compounds were present at concentrations as follows: soyasaponin I—1.05 µg/mL; daidzein—0.48 µg/mL; genistein—0.27 µg/mL; and glycitein 0.06—µg/mL. Additionally, at MIC 4096 µg/mL, the growth of the rest of the tested strains was inhibited, including the bovine mammary gland isolate *S. aureus* CCM 6188 (MSSA); the *S. aureus* strains ATCC 33591 (MRSA), ATCC 43300 (MRSA), and ATCC BAA 976 (MRSA); and the clinical isolates EMRSA-15 and SA 3 (both MRSA). At the concentration of MED of 4096 µg/mL, bioactive compounds were present at the following concentrations: soyasaponin I—2.11 µg/mL; daidzein—0.97 µg/mL; genistein—0.54 µg/mL; glycitein 0.12—µg/mL.

Bacterial growth control testing in the presence of penicillin G (synthetic benzylpenicillin) showed MIC values to be consistent with the antibiotic susceptibility patterns of the tested *S. aureus* strains. The lowest values were detected in methicillin-sensitive *S. aureus* strains. The most sensitive were *S. aureus* CCM strains 4442 and 6188 with the MIC of 0.01563 µg/mL, followed by the strain ATCC 29213 with an MIC of 0.125 µg/mL. Methicillin-resistant strains showed MICs of penicillin G higher than 0.125 µg/mL. The respective MIC values for MRSA strains were 1 µg/mL (SA 2), 2 µg/mL (SA 3), 32 µg/mL (ATCC BAA 976 and EMRSA-15), 64 µg/mL (ATCC 43300), and 512 µg/mL (ATCC 33591). Moreover, it was observed that DMSO did not affect the antibacterial properties of the MED as it produced no antibacterial action toward the tested *S. aureus* strains at a concentration of 10,000 µg/mL, which was not exceeded during the sample preparation.

The use of MTT dye after the in vitro broth microdilution testing confirmed the obtained values from MIC determination (data not shown).

### 3.3. Growth of Staphylococcus Aureus Strains with Various Concentrations of MED

**Figure 3:**
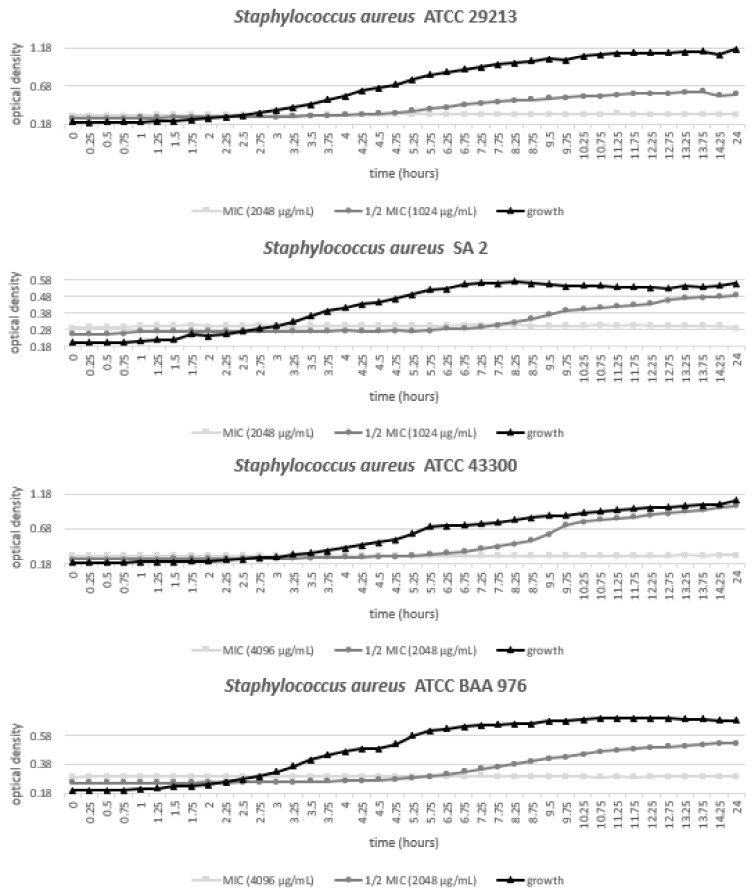
Growth curves of chosen *Staphylococcus aureus* (*S. aureus*) strains at two concentrations (minimum inhibitory concentration, MIC; half of minimum inhibitory concentration, ½ MIC) of methanolic extract of doenjang (MED).

To evaluate the dynamic interaction between the MED and the tested bacterial species, an analysis of growth by modeling the curves for four *S. aureus* strains, namely ATCC 29213, ATCC 43300, ATCC BAA 976, and the clinical isolate SA 2, was performed. From the measured values of eight concentrations, growth curves of MIC and ½ MIC were drown only. The graphical evaluation of bacterial growth in Figure 3 reveals the antibacterial manner of action. The MED used at MIC against the chosen *S. aureus* bacterial strains caused the growth curve to be linear since the beginning of the measurement. On the other hand, the application of the MED at ½ MIC caused the deceleration of the bacterial growth as the lag phase of the growth took longer when compared to the MED-free control growth curve.

## 4. Discussion

Bacterial antibiotic resistance is a multifactorial issue influenced not only by the consumption of antibiotics in human medicine but also strongly by the administration of antibiotics to livestock [57]. According to Van Boeckel et al. [58], the consumption of antibiotics in animal production is predicted to increase by 67% between 2010 and 2030, especially due to the increase in population in middle-income countries, where the intensification of animal production is expected. For this reason, the emergence of livestock-associated strains of *S. aureus* that are known to be transmittable to humans [59] is a relevant issue for both human and veterinary medicine [60], and efforts on an international level, such as the “One Health” approach, have been implemented [61]. Although bacterial antibiotic resistance is recognized as a worldwide threat, the attitude to this problem in terms of antibiotic administration to livestock for growth promotion and their prophylactic properties is not uniform across the world [62]. The strictest rules are applied in the European Union, where the use of antibiotics in livestock is permitted for therapeutic use only [63]. These circumstances are thus implicated in the present intense research for alternative antibiotic substances that can be used in animal breeding, with natural compounds being a valuable source [64]. 

As described previously by various authors [31,38,41,65,66], the antibacterial activity of soy isoflavones is induced by affecting the integrity of the cell wall and membrane, by the inhibition of the respiratory metabolism and protein synthesis of the bacteria, and, most importantly, by the prevention of nucleic acid synthesis by influencing topoisomerase I and II or through topoisomerase IV. According to Wang et al. [28], soybean isoflavone extracted from defatted soybean meal completely inhibits the pBR322DNA unwinding mediated by topoisomerase I and topoisomerase II in *S. aureus* ATCC 26112 at a soy isoflavone concentration of 6400 μg/mL and could denature the plasmid DNA at a soy isoflavone concentration of 12,800 μg/mL. These concentrations are up to 6.25 times higher than the MICs of the MED tested in this study. Such a result supports the idea that due to the fermentation processes happening during doenjang production, the MED contains higher levels of antibacterial isoflavones (aglycone form) than the soybean isoflavone extracted from defatted soybean meal does [36]. Verdrengh et al. [38] showed that genistein, a soybean isoflavone, shows an ability to inhibit the growth of three MRSA strains up to 26-fold at a concentration of approximately 27 μg/mL, which is 76–151 times lower than in the case of MED. On the other hand, pure daidzein, which has been detected as a main isoflavone of the MED, is believed to express lower antibacterial activity when compared to genistein [41,67]. Moreover, Dhayakaran et al. [68] detected no antibacterial activity of soy isoflavones isolated from low-fat soy flour by microtiter plate assay at a concentration of 100 μg/mL against one MRSA strain. As the content of genistein (148.56 ng/mg) was comparable to that in the MED (131 μg/mL), the results are in accordance, since Dhayakaran et al. did not evaluate higher concentrations of their soy isoflavone.

According to the results of this study, mixtures of isoflavones and other bioactive compounds obtained by extraction from soybean products possess lower antibacterial efficiency when compared to the activity of pure isoflavones described in the abovementioned studies [38,65]. Extracts of isoflavones from different sources may contain not only the free or aglycone forms of isoflavones but also conjugated or glycosidic forms, which are generally known to exhibit decreased biological activity [69]. Moreover, extracts from samples consisting of isoflavones may comprise other biologically active substances that can hinder the antibacterial activity of the isoflavones themselves. As mentioned previously, soybeans, their products, and extracts are known to contain other bioactive molecules, which can possibly interact with isoflavones and decrease their antibacterial function. As reviewed by Xu et al. [70], soy isoflavones not only show mutual interactions, but their activity can be influenced by other compounds such as vitamins, trace elements, chemotherapeutics, and phytoestrogens. Thus, animal health may be influenced in both a positive way (e.g., hypocholesterolemic effect of a soy diet in rats possibly induced by interactions between the isoflavone and soy protein [71]) and a negative way (e.g., stimulation of the growth of breast cancer cells in culture and uterine enlargement in rodents [72]). Moreover, even though DMSO did not affect the bacterial growth in this study, its contribution in combination with the multiple compounds present in the extract cannot be excluded. Furthermore, the differences between the results of the previously mentioned studies reporting the antibacterial activity of isoflavones against *S. aureus* could be caused by various factors influencing the experiment, such as the inconsistent ways of antibacterial testing, including strains used, culture conditions, and methodology; different procedures of obtaining isoflavones; and importantly, the distinct constitution and concentration of the tested isoflavones in the tested extracts. To minimize the influence of antibacterial activity testing, the microdilution broth method was used in this study, which is a standardized assay recommended by the CLSI and in use worldwide [54]. Hummelova et al. [40] defined the hydroxyl groups at the C-5, -6, and -7 positions as crucial to the antibacterial action of plant isoflavones and their metabolites and evaluated trihydroxyisoflavones as antibacterially active. The importance of the isoflavone structure can possibly clarify the results of the study presented, as only genistein (5,7,4′-trihydroxyisoflavone; average content 131 ng/mg), displays a structure determining the antibacterial activity compared to daidzein (7,4′-dihydroxyisoflavone; average content 236 ng/mg) and glycitein (7,4′-dihydroxy-6-methoxyisoflavone; average content 29 ng/mg), which do not fulfill the condition of being trihydroxyisoflavones. Furthermore, glycitein has a methoxy group in its structure, which is proposed to decrease the effect of antibacterial activity [73]. 

The results obtained in this study show that the MICs of the MED are above the levels generally recognized as effective for practical applications. Concerning the dosage of MED for animals, there are no data available since it has not been used in animal nutrition yet. Nevertheless, as stated above, in addition to the antibacterial action, substances occurring in the MED possess different biological activities that can be beneficial to animal health and production. It has previously been proven that genistein induces cytotoxicity in both tumoral and non-tumoral cells only at supraphysiological levels (≥13.5 µg/mL) [74]. According to Han et al. [75], the half maximal inhibitory concentration (IC50) of daidzein in different human cancer cell lines is ≥15.18 µg/mL. Moreover, glycitein has no cytotoxic effects in various normal human cells (including normal stomach cells), even at a concentration of 28.43 µg/mL [76]. In this study, it was observed that the content of isoflavones occurring in the MED did not reach sufficient levels to cause toxicity to cell lines, even at the concentrations that exerted an antibacterial effect against the tested *S. aureus* strains. Considering the average amounts of the main identified isoflavones (Table 2) at the MICs of the MED (2048–4096 µg/mL), daidzein, genistein, and glycitein were present at respective concentrations of 0.48–0.97, 0.27–0.54, and 0.06–0.12 µg/mL; thus, administration of the MED to animals even at its MIC can be considered safe.

To the best of our knowledge, there is only limited information on the antistaphylococcal properties of MED, but the separate isoflavones of soybean in aglycon form are known to show an antibacterial effect [31,38,39]. In addition to the antimicrobial action, isoflavones and soyasaponins occurring in the MED are known to be modulators of other biological processes whose effects can possibly contribute to animal health [47]. Hence, administering the MED in the form of a feeding additive to different livestock species may have a positive impact on animal health, with various advantages resulting from the production of biologically active substances by the fermentation processes during doenjang manufacturing [77]. Wocławek-Potocka et al. [78] summarized the advantages of feeding materials with isoflavones, such as genistein and daidzein, which have been addressed as preventive factors for cancer risk and cardiovascular diseases and as anti-obesity, neuroprotective, and osteoprotective agents. However, it is necessary to mention the estrogenic action of soy isoflavones that can negatively influence animal production. According to Kaminska et al. [79], genistein and daidzein at concentrations of >2.5 µg/mL are able to significantly decrease basal and ACTH-stimulated cortisol and corticosterone secretion by porcine adrenocortical cells harvested during the luteal as well as the follicular phase of the estrous cycle. Nevertheless, genistein and daidzein at the MICs of MED (2048–4096 µg/mL) were present at respective concentrations of 0.27–0.54 and 0.48–0.97 µg/mL. Due to this fact, the intake of isoflavones in MED during its feeding to animals even at the MIC should not exceed levels that influence the estrous activity of the livestock.

In summary, this in vitro study revealed an antistaphylococcal effect of the MED. Thus, this study proposes the MED as an alternative to in-feed antibiotics for livestock as the antibacterial action was also proven for *S. aureus* strains that appear in dairy cows. The addition of the MED to the daily ratio of food-producing animals can possibly serve as a source of natural antibacterial substances such as isoflavones. We can hypothesize that due to the various biological effects, soybean isoflavones occurring in the MED may have a positive impact on livestock production. However, these assumptions need to be confirmed first by determining the antimicrobial activity of the tested extract for other bacterial species —not only pathogenic but also health-promoting bacteria, such as *Lactobacillus* spp. and *Bifidobacterium* spp. —and secondly, by in vivo studies on both polygastric and monogastric animals, as the metabolic pathways can either increase or decrease the antibacterial activity of soybean isoflavones.

## 5. Conclusions

In this study, it has been experimentally proven in vitro that the methanolic extract of the Korean soybean fermented product doenjang in higher concentrations shows antistaphylococcal properties. The antibacterial activity of the MED was exhibited in all tested strains including MSSA and MRSA and in animal clinical isolates (bovine mastitis and mammary gland isolates). Measurement of the growth parameters of the chosen *S. aureus* strains with the MIC of the MED confirmed its antibacterial mode of action, suggesting the MED as a promising source of antibacterial compounds in animal nutrition. According to the UHPLC analysis, the substances responsible for the antibacterial action of the tested extract are supposed to be the isoflavones daidzein, genistein, and glycitein, which were found in the MED at countable amounts. Moreover, the MED is a rich source of soyasaponin I, which possesses various biological activities, including anti-inflammatory and anti-hypertensive properties. However, further in vitro and in vivo studies on the effect of the MED are needed to examine the effects on animal health and production.

## Figures and Tables

**Table 1:** Bacterial strains and their specification.

Bacterium	Strain	MSSA/MRSA	Specification
*Staphylococcus aureus* subsp. *aureus*	ATCC 29213	MSSA	Standard strain for CLSI antimicrobial susceptibility testing
	ATCC 33591	MRSA	SCC*mec*: Type III *spa* type Ridom: t037 *spa* type Kreiswirth: WGKAOMQ *pvl* gene amplification: negative
	ATCC 43300	MRSA	Oxacillin-resistant SCC*mec*: Type II *spa* type Ridom: t007 *spa* type Kreiswirth: WGKKKKAOM *pvl* gene amplification: negative
	ATCC BAA 976	MRSA	Tracheal aspirate Clinical specimen Isolated March 2003
	CCM 4442	MSSA	Bovine mastitis isolate (Czechia) Production of β-hemolysin Atypical strain Phosphatase and clumping factor negative
	CCM 6188	MSSA	Bovine mammary gland isolate Loss of hemolysins production
	EMRSA-15	MRSA	Human origin Epidemic strain Oxacillin-resistant Penicillin-resistant
	SA 2	MRSA	Human origin Clinical drug-resistant isolate Oxacillin-resistant Penicillin-resistant Gentamicin-resistant Tetracycline-resistant
	SA 3	MRSA	Human origin Clinical drug-resistant isolate Gentamicin-resistant Penicillin-resistant

ATCC: American Type Culture Collection; CCM: Czech Collection of Microorganisms; MSSA: methicillin-sensitive *Staphylococcus aureus*; MRSA: methicillin-resistant *Staphylococcus aureus*.

**Table 2:** Amount of isoflavones and soyasaponins in the methanolic extract of doenjang (MED), assessed by UHPLC/MS analysis.

Bioactive Compound	Average Content ± STD (ng/mg)	Precision (RSD)	LOD (ng/mg)	LOQ (ng/mg)
Soyasaponin I	515.40 ± 0.46	0.089%	0.19	2.91
7,4′-dihydroxyisoflavone (daidzein)	236.30 ± 4.85	2.05%	0.22	0.73
5,7,4′-trihydroxyisoflavone (genistein)	131.23 ± 2.32	1.77%	0.33	1.11
7,4′-dihydroxy-6-methoxyisoflavone (glycitein)	29.00 ± 0.26	0.91%	0.14	0.47
7-hydroxy-6-methoxyisoflavone	tr.	-	0.08	0.25
7-methoxyisoflavone (methoxyisoflavone)	tr.	-	0.09	0.30
7-hydroxy-4′-methoxyisoflavone (formonetin)	tr.	-	0.10	0.34
6,7,4′-trihydroxyisoflavone (demethyltexasin)	tr.	-	0.22	0.75
7,3′4′-trihydroxyisoflavone	tr.	-	0.24	0.80
7,4′-dimethoxy-5-hydroxyisoflavone (dimethylgenistein)	tr.	-	0.16	0.52
7-hydroxyisoflavone	ND	-	0.10	0.34
5,7,4′-trimethoxyisoflavone	ND	-	0.06	0.21
6,7,4′-trimethoxyisoflavone	ND	-	0.08	0.27
4,7,8’-trimethoxyisoflavone	ND	-	0.08	0.27
5,7-dihydroxy-4′-methoxyisoflavone (biochanin A)	ND	-	0.23	0.75
6,4′-dimethoxy-7-hydroxyisoflavone (afrormosin)	ND	-	0.08	0.26
6,7-dimethoxyisoflavone	ND	-	0.09	0.29
7,4′-dimethoxyisoflavone	ND	-	0.08	0.28
7,12-dihydroxycoumestan (coumestrol)	ND	-	0.93	3.12

ND: not detected (below LOD); tr.: traces (below LOQ but greater than LOD); STD: standard deviation of five repeated measurements of the extract; RSD: relative standard deviation of five repeated measurements of the extract; LOD: limit of detection; LOQ: limit of quantitation.

**Table 3:** Antibacterial effect of the methanolic extract of doenjang (MED), penicillin G, and dimethyl sulfoxide (DMSO) on *Staphylococcus aureus* strains evaluated by the microdilution broth method as minimum inhibitory concentrations (MICs).

*Staphylococcus aureus* Strain	MIC (μg/mL) ^1^		
	MED	Penicillin G	DMSO
ATCC 29213 *	2048	0.125	>10,000
ATCC 33591 ^†^	4096	512	>10,000
ATCC 43300 ^†^	4096	64	>10,000
ATCC BAA 976 ^†^	4096	32	>10,000
CCM 4442 *	2048	0.01563	>10,000
CCM 6188 *	4096	0.01563	>10,000
EMRSA-15 ^†^	4096	32	>10,000
SA 2 ^†^	2048	1	>10,000
SA 3 ^†^	4096	2	>10,000

^1^ Mode of three analyses, each performed in triplicate; * methicillin-sensitive strain; ^†^ methicillin-resistant strain.

## Data Availability

The data presented in this study are available within the article and the Supplementary Materials.

## Supplementary Materials

The following are available online at https://www.mdpi.com/article/10.3390/ani11082319/s1. Table S1: MS settings. Table S2: Characteristics of compounds determined by UHPLC/MS HRAM in the methanolic extract of doenjang sample. Figure S1: Fragmentation spectra (HRAM-MS/MS) of detected isoflavone glycosides (positive mode). Figure S2: Fragmentation spectra (HRAM-MS/MS) of selected soyasaponins (positive mode).
