# Supplementary material for: In Vitro Antibacterial Effect of the Methanolic Extract of the Korean Soybean Fermented Product Doenjang against Staphylococcus aureus
Source: Animals (Basel). 2021 Aug 5;11(8):2319. doi: 10.3390/ani11082319 (PMC8388408; doi:10.3390/ani11082319)
Supplement: Supplementary file 1 [file animals-11-02319-s001.zip › animals-1316404-supplementary.pdf]

Supplementary material

Figure S1. Fragmentation spectra (HRAM - MS/MS) of detected isoflavone glycosides (positive mode).

MS/MS spectra of Genistein-hex (probable genistin).

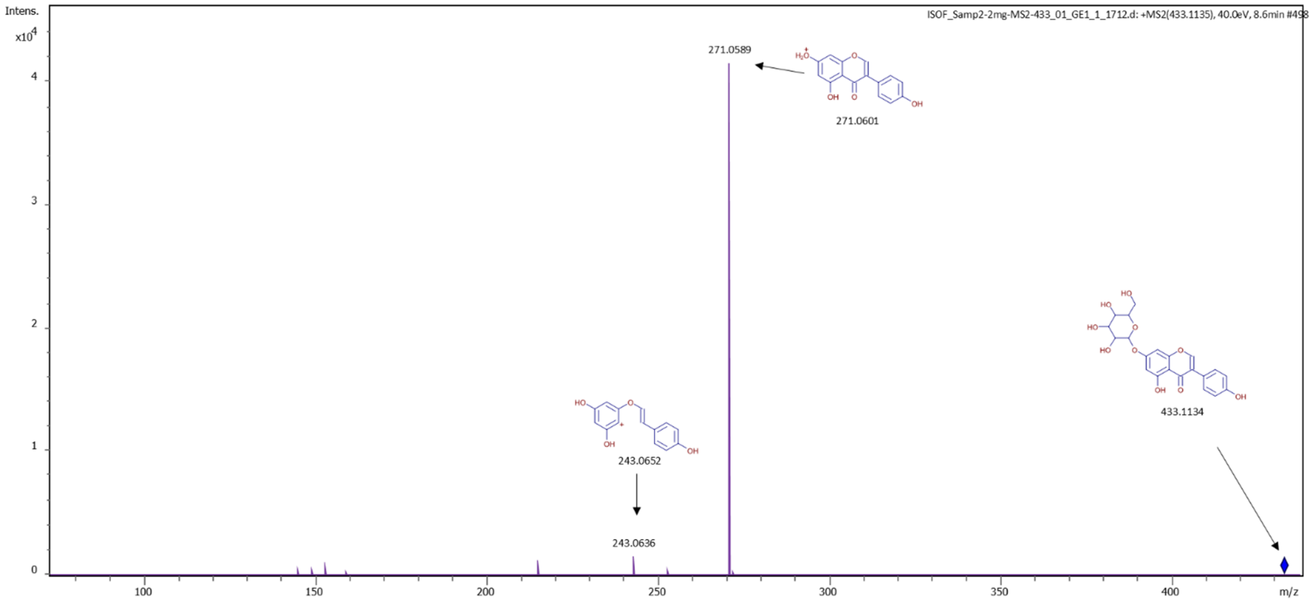

MS/MS spectra of Glycitein-hex (probable glycitin)

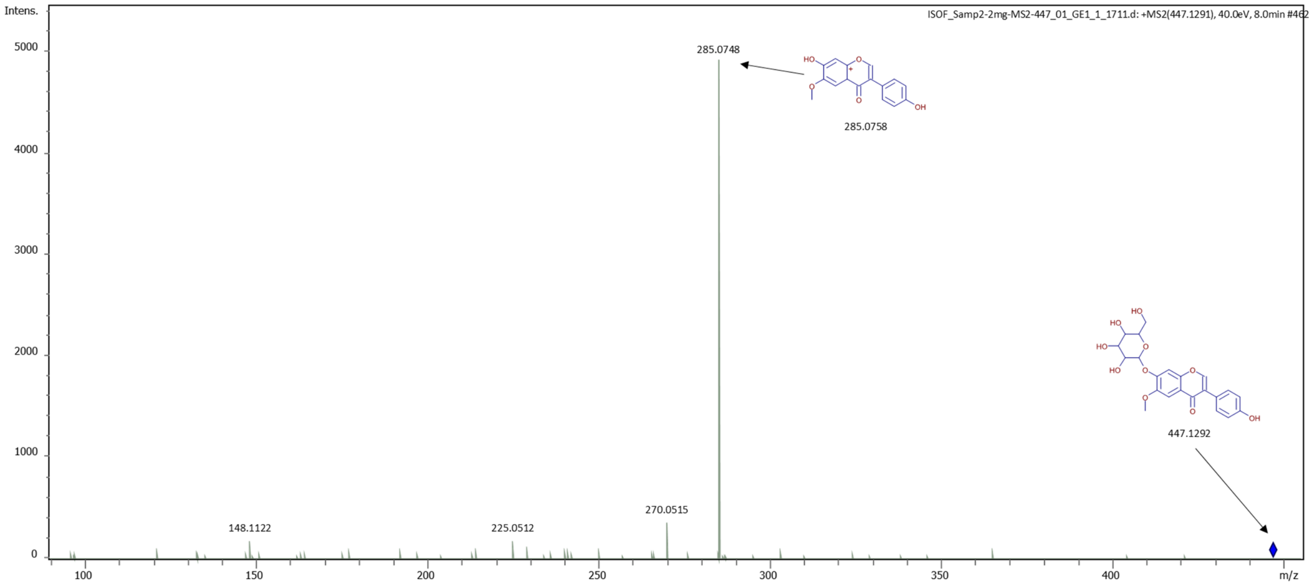

MS/MS spectra of Daidzein-hex (probable daidzin)

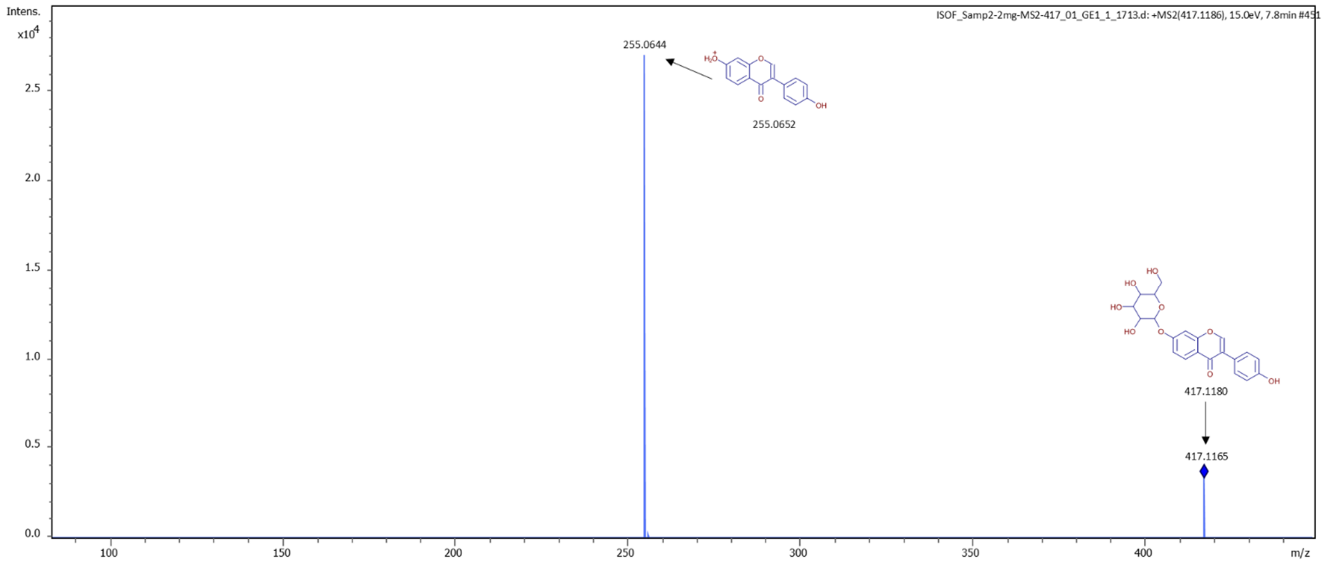

UHPLC/MS extracted chromatogram of detected isoflavone glycosides.

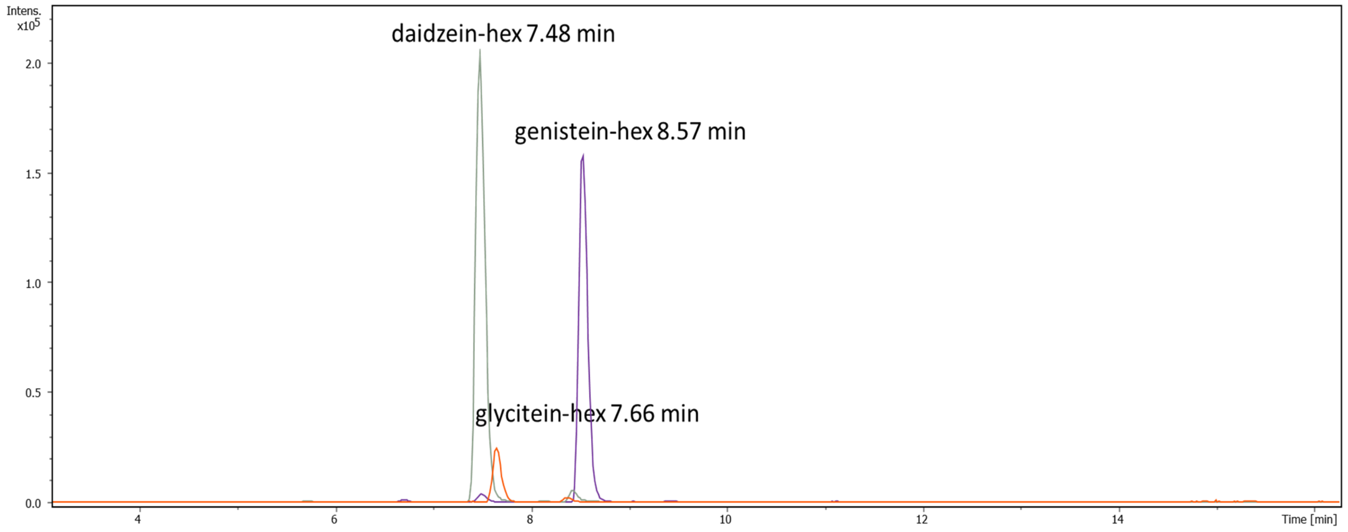

Figure S2. Fragmentation spectra (HRAM - MS/MS) of selected soyasaponins (positive mode).

MS/MS spectra of probable Soyasaponin II

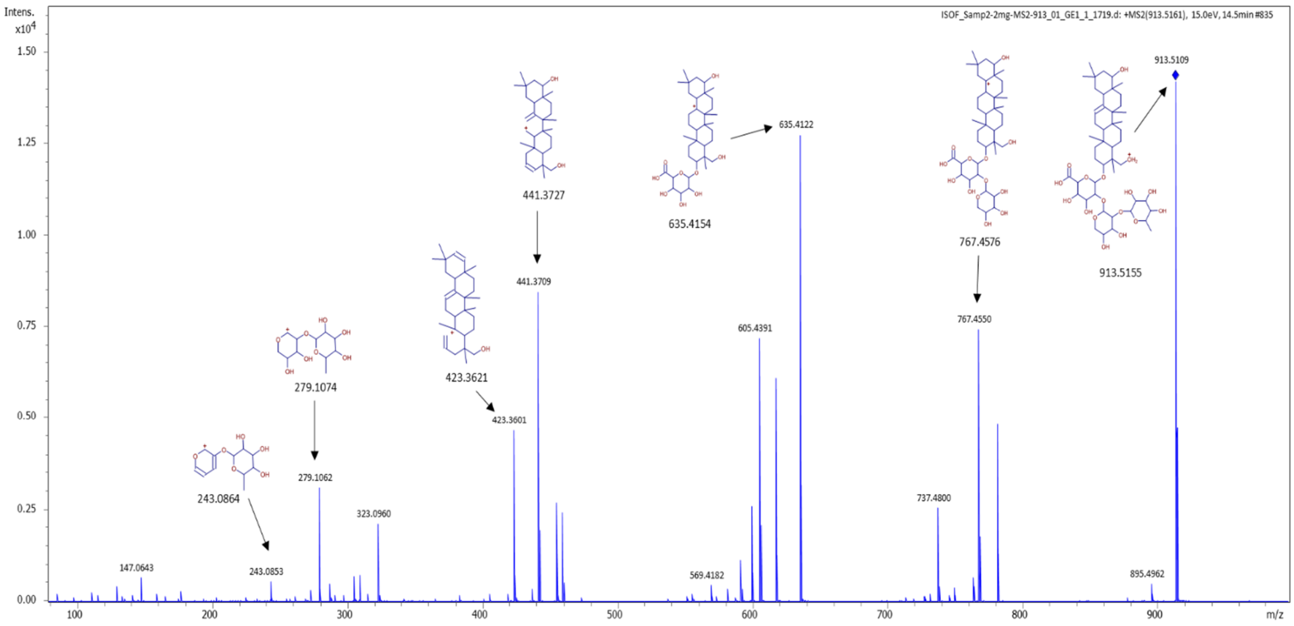

MS/MS spectra of probable Soyasaponin III

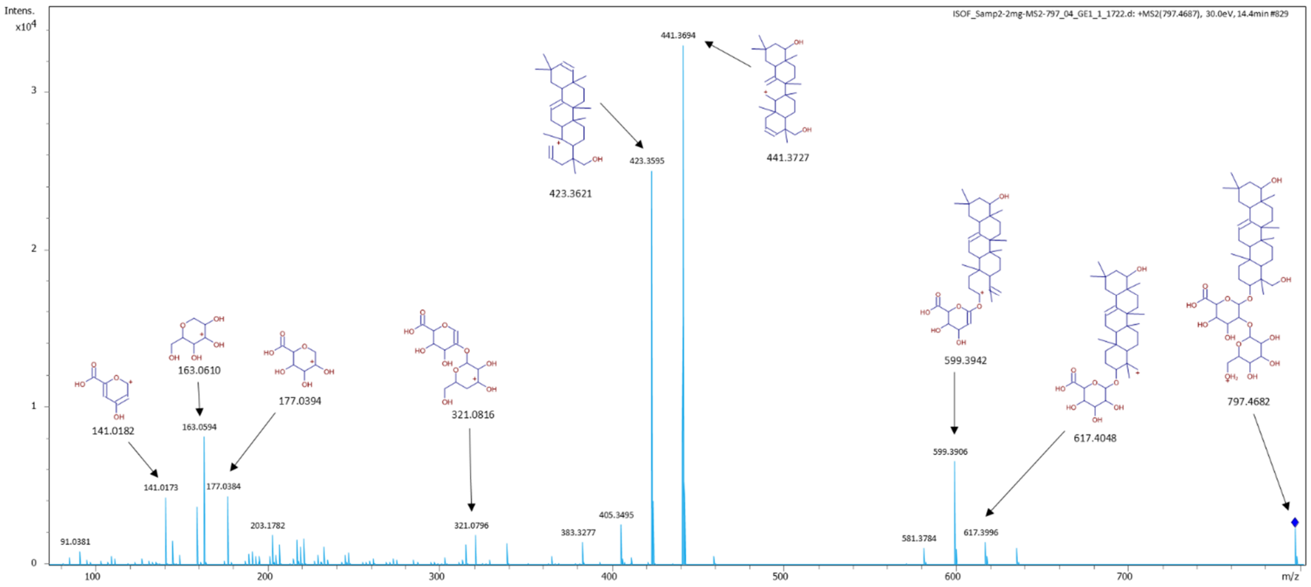

MS/MS spectra of probable Soyasaponin V

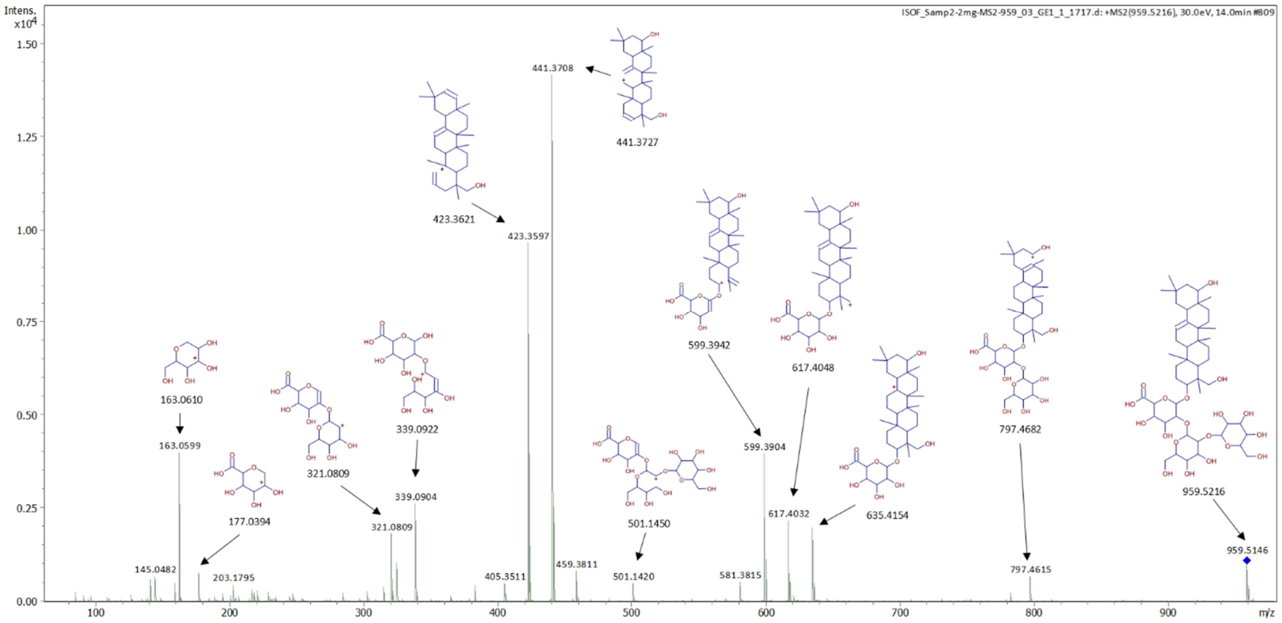

Table S1. MS settings.

Ion source settings:

end plate offset:	500 V
capillary voltage:	2 500 V
nebulizer gas:	2.0 Bar
dry gas:	5.0 L/min
dry temperature:	300°C

Acquisition settings:

mass range:	60 – 1 500 m/z
scan rate:	1 Hz
resolution:	> 60 000

Table S2. Characteristics of compounds determined by UHPLC/MS HRAM in the methanolic extract of doenjang sample.

Compound	Formula	$[M+H]^+$ calculated	$[M+H]^+$ measured	RT [min]
Isoflavones				
7,3'4'-trihydroxyisoflavone	C15H10O5	271.0606	271.0605	9.6
6,7,4'-trihydroxyisoflavone	C15H10O5	271.0606	271.0602	9.7
Glycitein	C16H12O5	285.0763	285.0759	10.3
Daidzein	C15H10O4	255.0657	255.0651	10.6
7-hydroxy-6-methoxyisoflavone	C16H12O4	269.0814	269.0795	11.2
7-methoxyisoflavone	C16H12O3	253.0865	253.0864	11.8
Formonetin	C16H14O5	269.0814	269.0809	11.7
Genistein	C15H10O5	271.0606	271.0602	11.7
7,4'-dimethoxy-5-hydroxyisoflavone	C17H14O5	299.0919	299.0907	13.2
Isoflavone glycosides				
Daidzein hexoside (daidzin)	C21H20O9	417.1186	417.1184	7.5
glycitein hexoside (glycitin)	C22H22O10	447.1291	447.1292	7.7
genistein hexoside (genistin)	C21H20O10	433.1135	433.1134	8.6
Soyasaponins				

Soyasaponin I	C48H78O18	943.5266	943.5258	14.3
Soyasaponin II	C47H76O17	913.5161	913.5150	14.5
Soyasaponin III	C42H68O14	797.4687	797.4675	14.4
Soyasaponin IV	C41H66O13	767.4582	767.4568	14.5
Soyasaponin V	C48H78O19	959.5216	959.5218	14.1
Soyasaponin VI	C54H84O21	1069.5583	1069.5604	15.0
